# Supporting elderly patients in strengthening their personal and spiritual health resources

**DOI:** 10.3389/fpsyg.2023.1237138

**Published:** 2023-11-10

**Authors:** Bogusław Stelcer, Aleksandra Bendowska, Dorota Karkowska, Ewa Baum

**Affiliations:** ^1^Department of Human Nutrition and Dietetics, Faculty of Food Science and Nutrition, Poznan University of Life Sciences, Poznań, Poland; ^2^Department of Clinical Psychology, Poznan University of Medical Sciences, Poznań, Poland; ^3^Department of Social Sciences and the Humanities, Poznan University of Medical Sciences, Poznań, Poland; ^4^Institute of Nursing and Midwifery, Collegium Medicum, Jagiellonian University in Krakow, Kraków, Poland

**Keywords:** spirituality, health resources, spiritual care, end of life care, gerontologic care

## Abstract

Care for the sick, including spiritual support is sometimes called holistic medicine. The term bio-psycho-social-spiritual model is sometimes used to describe this type of therapeutic approach patient-oriented medicine. This report indicates the importance of taking into account the spiritual aspect of life due to its beneficial impact on the coping resources activated and the patient’s well-being. Existential and spiritual issues are on the verge of new clinical and research interest in medicine, especially in gerontology, oncology, and palliative care. Clinicians focus not only on symptom control but also on spiritual and existential issues such as spirituality, hope, and meaning. This paper reviews the topic of spirituality in the context of illness and end-of-life care trying to define spirituality within the context of health resources of the subject. Spirituality is perceived in two of its main components: faith/religious beliefs and spiritual well-being. Especially this second construct is reviewed and described as a health resource.

## 1. Introduction

Spirituality, religion, and faith appear interchangeably in the scientific literature (Yeşilçınar et al., 2018). However, these concepts differ in their definition and meaning (Barber, 2019). Spirituality is there often misunderstood due to its deep associations with religion (Jones, 2018). Scientific literature provides a number of evidence proving the relationship between faith and spirituality and their impact on coping with life crises, illness, health, and well-being. Spirituality supports mechanisms for coping with difficult situations such as disease (Barber, 2019). Spirituality can support the adjustment process of people with diseases, by forming a meaning system that supports understanding of the cause and implications of the experience and that provides coping strategies. According to research conclusions, faith is a powerful factor promoting hope (Fowler, 2017). It should be noted that spiritual support and religious faith help maintain hope in difficult situations (Nedderman et al., 2010; Timmons, 2012). The aim of this article is to indicate selected psychological concepts referring to a person’s spiritual resources that allow him to cope with accumulated losses in late adulthood.

## 2. Spirituality as a personal health resource

Spirituality is currently a legitimate topic for discussion in the field of philosophy of medicine, psychology, bioethics, and related fields. Medicine has long abandoned the Cartesian dualism of the mind and body and its implications in favor of a more holistic research perspective (Swinton, 2001). Therefore, understanding the nature of the spiritual seems to be the key to grasp the concepts of health, well-being, and the quality of life, all firmly established in the medical literature (Orchard, 2001). In medical practice, the conviction has emerged that healthcare institutions can provide spiritual care as a rightful part of the healthcare package (Graber and Johnson, 2001; Klimasiński et al., 2017). Incorporating spiritual care into the healthcare system is becoming increasingly justified (Boscaglia et al., 2005). Along with the advances in research on mental well-being and the relationship between health and the quality of life, there is an enhanced understanding of the significance of the role that spirituality plays in well-being and health (Bożek et al., 2020). Simultaneously, there is a growing interest in health in relation with positive aging. Spirituality has been present in medical literature for no less than 2 decades, and it has been emphasized in papers from the fields of psychiatry, medicine, general practice, and nursing (McSherry, 2001; Culliford, 2002; Greenhalgh, 2002; Walsh et al., 2002).

From the existential point of view, spirituality is connected with the way in which the person can relate to something existing beyond them and through that relation find the meaning of life. The essence of spirituality is the relation of the *I* to something beyond. Spirituality is a term broader in scope than religion. Its ethical quality must be indicated—religion without reference to spirituality, let alone ethics, is not elevated, high, or valuable, but limited to a system of rules of conduct (Krzyżanowska-Łagowska, 2005). According to the historical Arthur Schopenhauer view, the Christian religion is essentially existentialist, which manifests itself through the concern for the good of others (Schopenhauer, 2017). The current understanding of religion relies on its perception as an element linking a human to the sacred domain. Religion is perceived one of the most significant aspects of spiritual life. At the same time, it is true to say that people who do not believe in a religious sense often have a deep spiritual life. There can be no spirituality without relating the self to something beyond it. The spirit can be perceived as a life-driving force, as it is its essence and meaning. Meaning can be found in many acts of everyday life, including love, hope, transcendence, and the search for harmony and well-being. Spirituality involves different perspectives, including psychological, transpersonal, and religious. It is fully communicated by all forms of expression present in cultural codes. Spirituality is a factor closely connected with an individual’s health and sanity. While conducting studies on the importance of the spiritual dimension at the end of life, Bryson (2004) formulated the following findings with regard to its nature:

- spirituality can be understood as an innate tendency to seek and give meaning;
- spirituality is present in worldly life;
- spirituality is brought to life and enhanced by transcendence;
- spirituality leads people to achieve unity, truth, good, and sympathy;

### 2.1. Spirituality from psychological perspectives

Spirituality is thus a tendency to seek meaning and relation with a force that remains beyond the person. It is a reference to something that remains outside the person giving him or her a sense of belonging to something greater than he or she. At times of crisis, pressure or everyday challenges, it allows the person to rely on a supporting frame of reference, to step beyond himself or herself, and find the spiritual assistance that will enable them to gain insight and give meaning to the experience they are struggling with (Frankl, 1992). A mark of spirituality is also the ability to seek and give meaning to the events encountered (Chojniak, 2003).

The introduction of terms related to the issues of spirituality and religiosity/religiousness to the humanities reflects the modern trend toward a holistic view on the development of the human personality in its physical, mental, and spiritual aspects (Gałdowa, 1999). One of such areas is health psychology, which grants spirituality a growing recognition (Heszen-Niejodek and Gruszczyńska, 2004). However, there is no unequivocal answer to the question of how to define spirituality. In the traditional approach represented by works of American psychologists, a semantic proximity between spirituality and religiosity is accentuated; indeed, a term has been coined which joins the two (*spirituality/religiosity*), equating them and treating as synonyms (Thoresen and Harris, 2002). Yet, other approaches treat the concept of spirituality as much more inclusive than religiosity, often regarded as more multifaceted and interdisciplinary.

One of the most comprehensive descriptions was provided by Tanyi (2002), who perceives spirituality as an individual search for meaning and purpose in life, not necessarily connected with religion. It is, however, related to the practices, values or beliefs that we choose for ourselves, which give meaning to our lives, inspire and motivate us to achieve genuine well-being. They make us feel a driving force inside, help us regain peace, hope, and faith. We feel joy and good energy that facilitates living a conscious life, accepting its imperfections, difficulties, and mortality (Stelcer, 2013). Such a deliberate distinction between spirituality and religiosity is advocated by other scholars, who narrow the concept of religiosity down to some external, institutionalized, superficial and ritualized practices, focused on preserving the rites and transmitting a doctrine (Chojniak, 2011). According to Socha (2000), “spirituality is a person’s *differentia specifica* and culture, in its broad sense, is a product of spiritual life” (own translation). Consequently, it can be seen that the notion of spirituality has a highly subjective, developmental and elusive character at the same time. In addition, the specific character of spirituality as an opportunity to develop throughout the whole life, including old age, is emphasized by Heszen-Niejodek and Gruszczyńska (2004), who point out that “spirituality is an attribute of every person” (own translation). Spirituality can be further specified in relation to areas in which it operates and manifests itself, such as, i.e., reflection, self-reflection, awareness and self-awareness, wisdom, faith, religion, meditation, contemplation, sensibility, a sense of strength and direct power, autonomy, and a sense of dignity and fulfillment; rational, emotional and perceptive sensitivity; worldview, the will to experience a valuable life filled with hope, plans, dreams, and visions (Von Humboldt et al., 2014).

In light of the above, the approach that insists on separating the concepts of spirituality and religiosity seems to be well-founded (Emblen, 1992; Dyson et al., 1997; Tloczynski et al., 1997; McSherry and Draper, 1998). The authors of this report understand spirituality as a principle of personal life, a search for and giving meaning; it brings to life a transcendent quality of a relationship with something external to the human. Spirituality is a term whose meaning is broader than that of religion. Nonbelievers do have a spiritual life; very often, their views and the underlying philosophical assumptions thereof are highly complex.

## 3. The spiritual realm and its connection with the health condition

A link between a person’s health condition and spirituality has also been observed. Initially, it was suspected that very religious people neglect their health in favor of spiritual practices, but it turned out that nothing could be further from the truth. A higher level of spiritual development corresponds to systematic use of the offered range of medical care services (Woodard and Sowell, 2001; Tuczyńska et al., 2022). Moreover, American epidemiological studies based on a very large population (21,204 people) demonstrated that the probability of death is 1.87 times higher in those who do not participate in any religious practices than in those who attend religious service at least once a week (Hummer et al., 1999). Larimore et al. (2002) claim that prayer, participation in religious rituals, or contact with clergy help the sick in the process of healing. Other researches show the importance that meeting patients’ spiritual needs is an integral part of end-of-life care. Supporting relationships should be a central focus of spiritual care for patients at the end of life (Clyne et al., 2022; Zapała et al., 2022).

The social aspect of participation in religious practices is mentioned by Pinker (2015), too, who explicitly highlights the fact that “faith brings people together in one place.” She also states that belonging to a religious group has a social, more altruistic and inclusive character that binds people together, and “coordinated rituals” give them “a sense of community without words.” The researcher also draws attention to the formation of long-term ties among followers of the same religion, who spend time together, but also naturally expect the principle of reciprocity to hold in mutual and multilateral relations. People are willing to help and they assume that in the face of an emergency, they will be given appropriate support, too. Religious people consider it a moral duty to take certain actions and are more inclined than atheists to act for the benefit of local community, make donations to charity, or do other prosocial activity (Bloom, 2008). Haidt (2006), American social psychologist who describes himself as a “Jewish atheist,” indicates another crucial role of divinity and sainthood in an individual’s life and calls it a third dimension, a moral dimension or a divine dimension. He states that having the element of divinity in us, we develop spiritually, we try to do good and do what is right, regardless of the degree of religiosity or specific faith. This view is confirmed by Seligman (2002), who points to the causal connection between religiosity and a decidedly more active prosocial life. Many religions prohibit practices that can be harmful to one’s health and require followers to exclude all kinds of stimulants or actions which intensify the feelings of anger, aggression or regret experienced by an individual. They recommend fostering positive feelings and adopting an attitude based on mercy and moderation and advice taking overall positive actions (Seligman, 2002). Furthermore, studies in psychology produced solid evidence that cognitive strategies and spirituality plays a significant role in an individual’s life, which motivated researchers to examine further dependencies between the inner life and health condition in particular groups of patients (Krawczyk et al., 2021; Domaradzki, 2022).

### 3.1. Spirituality as a resource for coping with illness

One of the first fields of medicine in which these links were tested was psycho-oncology. Nowadays, the expansive development of this field of science allows for effective support with psychological methods of basic oncological treatment (Stelcer, 2008). A particularly prominent role in this respect is played by psycho-neuro-immunology, which directly exposes the relationship between the nervous system and immune system, and as a result, underscores the connection between a subjective health status and an objective healing process (Larson et al., 1998; Sulmasy, 2002, 2009). Anxiety, depression, or dejection adversely affect the functioning of the immune system, while positive emotions precipitate its recovery (De-Walden-Gałuszko, 2005). In dealing with a life-threatening disease, oncological patients most often adopt an adaptive strategy, called “positive re-evaluation,” which, with the emergence of a new life situation, brings a change in the current system of values, leads to giving a deeper meaning to one’s own existence and makes a person consciously experience each coming moment of life, which causes old resentments, disputes and disputes to lose their importance, and the “life-giving hope” grows to become a symbol (Visser et al., 2018). The importance of religiosity additionally increases in terminally ill patients, as it frequently is the expression of their spirituality (Piskozub, 2010). Assumptions concerning the development of spirituality in its broad sense in the face of illness turn out to be practical not only for oncological patients, but also in the case of many other groups of patients, including the chronically ill. The same is true of medical students and medical staff as well (Kozak et al., 2010; Domaradzki and Walkowiak, 2021). They need to learn how to be prepared to confront with the pain, suffering and death of the patient (Szczupakowska et al., 2021).

Similar studies were conducted, among others, on patients with rheumatoid arthritis (Wróbel and Majda, 2015). In addition, some researchers claim that there is a close relationship between the degree of religiosity or spirituality, and a healthy lifestyle and longevity (Koenig et al., 2001). They believe that the degree of religiosity spirituality of the patient’s life should be taken into account when making medical decisions as to the further therapeutic procedure; thus, the significance of a patient’s spiritual or religious life should be considered by the treating physician (Anandarajah and Hight, 2001; Larson and Larson, 2003; Klimasiński et al., 2022). Frankl (1992), an Austrian psychiatrist and Holocaust survivor, repeatedly pointed to the therapeutic value of patients’ religious beliefs, seeing them as a source of spiritual resources. Although at this point it should be noted that there is no uniform view on the existence of a link between spiritual life and religiosity and health status, as other researchers suggest that many of the results of studies assessing the relationship between religiosity and spirituality of life and the health of patients are subject to methodological errors, and in practice there are few objective studies available demonstrating the link between the degree of religiosity or spirituality and the health of patients (Sloan and Bagiella, 2002). They believe that in the absence of irrefutable evidence concerning the link between spirituality and/or religiosity and the condition of a patient with regard to the prognosis of the progression of the illness, taking these assessments into consideration during a medical procedure is not justified.

Undoubtedly, however, the factors that help in coping with an illness are the individual predispositions of a person to react in specific life situations. They are referred to as resources and can be biological, psychological, social, or spiritual in nature (Borys, 2010). They trigger emotions that are natural “catalysts” of changes, releasing positive power and the desire to overcome difficulties. Health resources, which are of great importance to patients, are recognized as certain individual predispositions that a person, consciously or not, uses to improve their health or their own behavior. We derive internal health resources from our psyche. Personality aspects, such as temperament, play an important role, but also self-acceptance, a sense of identity and agency, a sense of control and self-control, the degree of resourcefulness as well as achieving goals and solving difficulties. Our ability to maintain behavioral control, i.e., personal influence on external factors, is crucial in perceiving our own agency while taking action to create a changing reality.

### 3.2. Autonomy as a personal resource

In line with a holistic view of man, subjectivity includes the somatic level, social level, and one that refers to the natural environment creating the environment in which the individual lives. Therefore, any attempts at proper understanding of spirituality should be made in relation to all the above-mentioned spheres. Disturbance of the harmony between them results in irregularities. An individual cannot function properly in the absence of adequate expression of one or more of these spheres of self-expression. Diseases accompanying old age dramatically destroy the psychosomatic integrity of an individual, causing the mind and the body to function detached from natural harmony. In light of this fact, psychological support given to an elderly person should aim at restoring the harmony between the three above-mentioned spheres, allowing them to regain well-being, strengthen their psyche and the sense of unity with your important ones and with the surrounding. The intensification of questions about the spiritual sphere appears particularly in the context of caring for people in the late years of life (Stelcer, 2013).

Existential crisis causes forceful questions to arise about the meaning of life, death, and the preceding suffering that accompanies chronic illness. One of the forms of constructive responses to this complex crisis is openness to the world, to full experience and living. Opening up to the world, to the sensations that this experience brings, is the first step to reflection on the spiritual dimension of existence (Stettbacher, 1993). In this sense, deep spirituality is strongly connected with and embedded in the real world, and flows from it. A person who is in good contact with reality, with facts and material beings will not lose him or herself in illusory metaphysical spaces and misconceived mysticism. Surprising as it may sound, Jung’s texts express a conviction that in order to enter metaphysical spaces and not to lose oneself in speculation, one must have good contact with the real world (Jung, 1989).

According to Walton (1996), it is important to see spirituality in the context of three interdependent dimensions. The violation of the harmonious relationship between the concepts and contents they refer to in terms of meaning, as well as the weakening or breaking of ties with external factors, some higher power, nature, with oneself or one’s own soul, lead to despair, to perceiving life as meaningless and oneself as a being helpless in the face of world events. In this context, spirituality can be understood as a special type of bond of an individual with other people and a transcendental being, accompanied by mutually intertwined interactions (Burkhardt, 1989). When people face difficulties or crises, such as old age, disease, and impending death, they strive to rediscover their relationship with others and with the God they believe exists. Spiritual relationships created in this way can be a great source of psychological comfort, providing healing energy and strength to take on challenges (Haan, 1984). Norma Haan, a psychologist from University of California, Berkeley, offers a similar explanation, stating that along with advancing years, three personality features continually develop. These are: *gaining distance* (taking a step back, humor etc.), *self-trust* (productivity, following advice), and *emotional warmth* (positive attitude, affirmation, sympathizing, protection, and giving).

## 4. Selected concepts of aging well and spirituality

As beings endowed with spiritual life, people strive for fulfillment, finding their way toward a sense of self-realization and achieving harmony. Positive old age is characterized by concern about life accomplishments and a positive balance at the end of it (Murata, 2003). Japanese culture created the concept of *Ikigai*, whose multifaceted definition refers to a positive balance of one’s life. Aging well includes behavioral mechanisms that promote good health and longevity, enabling the elderly person to function optimally (Tanyi, 2002). Gerontology is a discipline that combines the achievements of many scientific disciplines. Its goal is to understand the aging process. It covers sociology, psychology and medicine, nursing, and many other disciplines. Consequently, there are many different models and concepts that define the rules of successful aging. Psychological theories provide guidance on how to identify, explain, and predict human behavior leading to a positive overall balance at the end of the life. Therefore, theories of positive aging should be able to identify successful models of aging and explain why it was successful and ended with a positive balance. There are many complementary models and theories of positive old age, which are most often described in gerontology textbooks. These are based around some basic ideas about good health, lack of disease, indicators of autonomy and independence, social activity, and no overwhelming concerns.

Rowe and Kahn (1997) offered a model which explicitly associates successful old age with good health, a lack of chronic diseases, retaining physical fitness and mental capacity as well as the ability to perform certain physical activity. The model’s default assumption is that longevity and health are the attainments of late adulthood. More specifically, successful aging was defined by Rowe and Kahn as freedom from disease or disease-inflicted disability, high levels of cognitive and physical functioning, and active involvement in daily life. This model connects the physiological, psychological, and social spheres, emphasizing the heterogeneity of the process of aging well. This conception combines three dimensions of aging:

- physiological: diseases, disability;
- psychological: the dynamics and nature of emotions, coping, and resilience;
- social: spirituality, social adaptation, and the role of social support.

Another conception of positive aging was proposed by Baltes and Baltes (1990), who developed a model based on preserving adaptation and compensation skills. They suggest that successful aging is a process of simultaneous selection, optimization, and compensation. When these processes occur properly, the elderly person can maximize their “efficiency” with regard to keeping in touch with the mainstream of life. This model does not directly address the issue of spirituality, but it neatly shows that the task of selection, compensation, and optimization is not only to minimize the losses accompanying the aging process, but also a spiritual journey into the future, which is part of the psychosocial adaptation process of a senior. The approach to adaptation to old age is based on the belief that a person throughout his or her life encounters situations that force them to draw on their resources or to find other ones. Successful aging is perceived as maximizing positive and minimizing its negative effects; it also requires a person to focus on the more significant areas of life while withdrawing from the less significant ones. Adaptation to old age involves two mechanisms: *selective optimization*—that is, choosing these capacities (selection) which can be maintained and enhanced (optimization), while resigning from keeping equally high levels of functioning in other areas; and *compensation*—that is, developing means to compensate or replace the functions which were the most affected.

### 4.1. The theory of gerotranscendence

Interesting solutions were included in the so-called theory of gerotranscendence, formulated by Swedish gerontologist Tornstam (2005), who suggested that human development and maturing into old age are connected with a turn to transcendence. Referring to Carl Gustav Jung’s concept of individuation, Lars Tornstam assumes that gerotranscendence is the final stage of the naturally occurring progression toward maturity and wisdom. The theory of gerotranscendence presents achievable, positive aspects of old age, preceded by a harmonious life free from tensions. According to the assumptions of this theory, a person entering the late years of life redefines three dimensions of his or her existence: the self, relationships with the significant ones, and the experience of one’s own existence, which in this theory is referred to as the experience of the cosmic level. Surprising as it may seem, the theory of gerotranscendence evolved on the grounds of a lack of acceptance for and the marginalization of disengagement theories in gerontological literature. The key to understanding it is placing emphasis on shifting the area of activity from the external environment in favor of transformation toward enriching the inner world of the individual. It can be viewed as a theory of changes in consciousness late in life. The approach adopted by Thornstam derives inspiration from Zen Buddhism as well as phenomenological philosophy. In his view, the notion of gerotranscendence has a lot in common with the term *wisdom*. The dynamic process of a shift to gerotranscendence assumes the following changes in a person life (Tornstam, 1997):

- a growing awareness of cosmic unity;
- changing perception of the importance of time, space, and things;
- changing the way of assessing life and death and a decreasing fear of death;
- a growing sense of belonging to the past and future generations;
- a decreased interest in superfluous social interactions;
- a decreased interest in material things;
- lower egocentricity;
- spending more time on all forms of “meditation.”

Gerotranscendence reflects the final stage of a person’s life as a phase of natural progression into maturity and wisdom, from a perspective that accounts for change and development. Oldness is presented as a more contemplative dimension of old people’s lives.

### 4.2. Erik Erikson’s theory of psychosocial development

Erik Erikson’s theory of psychosocial development enjoys great popularity and coverage. According to this concept, old age, which appears after the generativity phase, is the last period in life that allows an individual to fulfill himself or herself (Erikson, 1993; Erikson and Erikson, 1998). An old person does not have this opportunity if he or she does not go beyond individual narcissism, has not been in an intimate relationship, and did not lead an active life in middle adulthood. Discussing developmental commitments of late adulthood, Erikson concentrated on the issue of psychological integrity (Scheck, 2014). This, he argued, cannot be achieved by an individual who did not live actively, going beyond individual narcissism, and never had an intimate relationship. The polar opposite of integrity is despair. Wisdom arises when an individual reflects back on life and is unable to positively assess its accomplishments (Erikson and Erikson, 1998). He or she cannot feel integrity with the effects of life, with what they leave behind. In Erikson’s approach to the final stage of life, an individual achieves psychological integrity which is a consequence of the previous stages in life, each of which involves corresponding developmental tasks. Erik Erikson notices that only those who developed the ability to take care of people and things, to cope with failures but remember moments of triumph, who continued to build their lives, can reap the rewards of the earlier stages and integrate with this sort of accomplishment (Erikson and Erikson, 1998). This is the real conclusion of old age. Old age can be and is perceived as the sum of life wisdom.

Considering the whole thing in psychological terms, without integrity in late years, one is left with a feeling that their life has been wasted and has not led to a point that would seem to matter. Without integrity in adulthood, a person has the impression that they have been living their life without a goal or direction, making space for despair. Moreover, without activity at its proper stage, it is difficult to see the order of the world. The way of thinking that stems from humanistic thought has been fully presented through the concept of the so-called “virtues” attributed with each developmental stage of personality as a resource. According to Erikson, these virtues are: hope, will, purpose, competence, fidelity, love, care, and wisdom—notions altogether typical of existential philosophers (Erikson, 1993; Stelcer, 2013). The suffering which the person does not understand, or which appears unexpectedly, poses a potential threat to his or her psychological integrity (Frankl, 1984). Viktor Frankl formulated his theses around the thought that it is not the enormity of suffering that is capable of destroying a person, but destructive is the pain, which is not able to be given meaning by the subject, is destructive (Frankl, 1986, 1992). Viktor Frankl himself experienced the absurd, meaningless war trauma of concentration camp. He is an excellent example that demonstrates the healing power of spirituality which enables an individual to resist dramatic experiences. In his opinion, spiritual relation makes it possible to survive even the most intense suffering (Frankl, 1984, 1992).

## 5. Spirituality in late adulthood

The experience of spiritual pain takes place in the present, but it also deprives the suffering person of hope for the future. The situation of a person aware of the approaching end of their life, as is the case in later years, forces one to reflect on the passing of time. Turning to the events of everyday life can have therapeutic value. Our lifespan, even if it is limited to a particular length, has great value, because something positive and significant may come into being if only for a short while (Cook et al., 2020). Everything that exists appears and takes shape in the context of time experienced by an individual. According to Heidegger, human life has a temporal structure which contains the present, and the present contains the past and the future. People turn to the future, accepting the existing past, which has already been. The past opens up possibilities which may come into existence in the future (Heidegger, 2010). Mount (2003) introduced a concept which he named “*an existential moment*,” which referred to a realization of one’s own fragility and finiteness of one’s existence. The awareness of the limited time of existence may become a factor of personal development. Distinguishing between being a human and being a person is extremely useful. Though every being is human in an anthropological sense, there are significant differences between individuals as to who they are in a personal sense.

A certain lifestyle may contribute to the increased intensity of the sense of pain and spiritual suffering, especially when there are no interesting phenomena for which there has never been a present. What is meant in this case is a life without interesting adventures, in other words—a boring one. In this sense, a reduced intensity of spiritual experience is caused by consumerism with its boring soap opera airing hours, commercials, shallow popular culture, and surrogate worlds filling the internet space while degrading genuine spiritual life and interpersonal relations. In a sense, this image corresponds to a mass man, so prophetically described in Gasset’s (2021) book “The Revolt of the Masses.” In contrast with the lifestyle of a “*self-righteous yonker*,” a life filled with interesting, meaningful experiences is a resource for a senior and every other person alike, protecting them from the fear of dying and death. In this case, considering a lack of opportunity for a long life, an interesting past full of experiences is an individual’s resource.

An understanding of the last phase of life similar to the above-mentioned was suggested by Erik Erikson in his concept of psychosocial development (Erikson and Erikson, 1998). The axis around which the developmental crisis of this stage of life revolves is the dialectical conflict between psychological integrity and despair. Late adulthood is a time of balancing and summarizing life achievements. According to this view, a mature man looks back on the course of his life and assesses the fruit of his life. The assessment concerns life achievements and its hedonistic and eudaimonic character that determine the sense of fulfillment (Deci and Ryan, 2008). At the last stage, the meaning and value of choices we made in life are affirmed and justified (Stelcer, 2013). In Eriksnon’s words, identifying with everything that followed from our life choices is the integrity of EGO. Feeling good about our choices is crucial in the process of accepting the inevitable end of our existence and facing the anxiety of imminent death. A positive balance is accompanied by a sense of accomplishment, satisfaction with the course of one’s life, and a sense of unity with its outcomes. A negative assessment of previous decisions and their consequences leads to a feeling of despair. This assessment is determined by a lack of acceptance of one’s life. Unfortunately, at an advanced age, it is too late to redirect your life and search for new existential solutions. A human at the final stage of his or her life is trapped, as on the one hand, they do not accept the state they are in, and on the other, it is too late to fix anything successfully. This is what the feeling of despair comes down to. If, however, one’s life followed a positive frame, the power of EGO is established, taking the form of a virtue appropriate to this phase that is wisdom. The achievement of this virtue confirms that a person has acquired the ability to live and be interested in it as an affirmed value (Erikson, 1993; Erikson and Erikson, 1998).

Late adulthood, multimorbidity, life-threatening conditions, and the prospect of death also mean an impending end to the ties between the patient and their significant others. The process of expiring relationships, along with the awareness of an approaching end of one’s life, is the most common source of spiritual pain. The loss of significant relationships filled with emotional bonds make incurable cancer patients experience spiritual pain connected with a loss of identity and the meaning of life. Psychological theories of attachment present an individual as a being that exists only in relation to significant others throughout the course of life. An individual’s identity is rooted in the relationships with others. The significant others give meaning to the ego which comes into existence and takes shape in the space created by attachment. An important feature of the spiritual dimension is its embeddedness in the sphere of social relations (Carroll, 1998). Spiritual search and questions about the meaning of life are carried out by giving a specific form to the relations linking the individual with the significant ones. In the space of social relations, an individual identity is born and created (Holmes, 2010). A person is not an abstract, but a real being; spirituality, along with a chance for healing and restoring mental balance, are possible thanks to, among other things, dialogue and important emotional bonds (Moberg, 2002). If a spiritual journey is an individual’s major task, its significance rises with the passing of successive years of life. Seniors knowingly get involved in this journey, which helps them maintain ties with people who can be of help to them.

Apart from the above indicated, another factor that increases spiritual pain is the loss of autonomy. The term encompasses not only the possibility of self-determination, but also intellectual independence and productivity, which are the key constituents defining a person in the subjective sense. An important feature of this phenomenon is an independent mental life connected with the ability to express subjective opinions and existing as a subject in contacts with others. However, there are a number of threats to such functioning. They include illness and old age which is characterized by the weakening of the functions performed by the body, the resulting disability, and dependence on the care of others. An ailing senior may experience spiritual pain connected with a sense of uselessness, worthlessness, and hopelessness of life, arising in the context of the loss of autonomy (Kissane et al., 2001).

### 5.1. Spiritual pain relief

The indicated categories, i.e., time perspective, quality of relationships, and a sense of autonomy, have a therapeutic value. Taking into account the above criteria, it is possible to formulate recommendations regarding the beneficial effects of spiritual care for seniors. Firstly, spiritual pain increases when a person realizes that their time on earth is dwindling and they can feel that the prospect of death robs them of the hope of existence in a distant future (VanderWeele, 2019). Sometimes religious beliefs that promise life after death are the solution. If an individual is able to find some form of “future after death,” be it reincarnation or afterlife, confirming this belief in conversations with therapists or clergy, they will definitely regain the meaning of life. For those who do not believe in any form of life after death, it is advisable that they are encouraged to appreciate every moment of life on earth, even if they are so transient. In this case, the therapeutic value lies in the kind of intervention which shows the profound meaning and positive nature of one’s time on earth, so that it is perceived as worth living. What is the most destructive is not the suffering itself, but such form of spiritual pain which cannot be made meaningful.

As far as the third indicated criterion is concerned; that is, a sense of autonomy, those behaviors of the senior person’s caregivers which strengthen his or her self-determination and decisiveness have therapeutic value. Autonomy factors include the opportunities that elderly people have to contribute to decisions affecting them, and the ability to formulate independent opinions and interpret the surrounding reality. So spiritual support includes discovering a future beyond death (the family will cope when I am not around anymore), retrieving others beyond death (I am proud of the children and grandchildren that I am leaving behind), and regaining autonomy in the face of death.

In Hermann’s studies on the spiritual needs of patients, a number of key aspects were identified. They included: the need for religion, the need for companionship, the need for involvement and control over the flow of events, the need to finish earthly business, the need to experience nature, and the need for positive outlook (Hermann, 2001). This study helped define specific directions for psychological intervention and provided guidance on how to organize care to meet the patient’s needs. The directions of psychological support that would provide for the spiritual sphere include the role of religious practices for sick believers, ensuring a quiet time for prayer, reading, listening to music, and visits by chaplains or social workers or other therapists. It is equally important to listen to the patient’s concerns in honest conversation, apply the so-called life review therapy, and enable the patient—despite their illness—to participate and be involved in family life through sharing information about daily matters and to support their need for connection with others.

The issue indicates the importance of the factor known in psychology as social support. Research on the relationship between spirituality and religiosity as well as obtained social support also includes a group of patients with end-stage renal disease. Finkelstein and collaborators noticed a clear relationship between a declared level of spirituality and selected domains of the quality of life, especially depression symptoms and the overall assessment of the quality of life by patients on dialysis (Finkelstein et al., 2007). Similar conclusions were drawn by Martínez and Custódio (2014), as in their study, a significant correlation between mental health and spiritual well-being was observed. The presence of the spiritual element in the patient’s life was the most important factor allowing to predict the state of his mental health (the presence of perceived fears, anxiety, sleep disorders, and psychosomatic disorders), and poor mental health was associated with a lower declared level of spirituality.

Patients’ spiritual life, social support received from the environment, and experiencing understanding and acceptance shown by the family, friends, and medical personnel, are all fundamental for the subjective perception of the quality of life by patients themselves. Those patients for whom spiritual life has greater importance also demonstrate an objectively greater ability to cope with illness. Consideration of the patient’s perception of the world and the recognition of his or her set of values play a central role in the therapeutic process.

According to Bryson (2004), there are three planes of relationships that shape an individual identity: relationship with oneself, with others and one in which the individual relates to his or her physical and cultural surrounding. In the above-cited essay on spirituality in palliative care, K. A. Bryson presents the method of teaching spiritual aspects of care developed by Neil McKenna, chaplain at Cape Breton Regional Hospital. The chaplain offered an interesting experiment to nurses and other hospice staff working with seniors. This clergyman used to begin his seminars with an analysis of the issue of compassion. In his opinion, compassion is the factor that brings people together, making them experience togetherness. However, it is important in this context to understand that we can share what we already have with others. Therefore, it is vital to start from one’s own spirituality. We cannot share with others something that we do not have, we do not know or understand. McKenna encouraged hospice nurses to investigate their own spirituality by trying to answer the following questions:

- What makes my life meaningful?
- What values and beliefs are fundamental in my life?
- How important is religion to me?
- How do religious postulates pertain to my spiritual sphere?
- To what degree would a life-threatening disease influence my daily functioning, my views, and the search for meaning?
- How can I introduce my own spiritual resources into my work?

The attempts to answer the above questions show us the directions of our own spiritual search. It is worth emphasizing that the awareness of one’s own spirituality and worldview facilitates a more effective aid response and enables a suggestion of important answers. Entering the spiritual realm enables at least partial identification with a specific set of values important to the patient (Kearney and Mount, 2000). Lack of spiritual activity and questions about meaning lead to an inevitable mental regression. A nurse, doctor, educator, psychologist, and any other member of the treatment team can help patients find meaning in their suffering by showing them compassion and encouraging them to ask important questions.

In a standard handbook of psychiatry in palliative medicine, Kearney and Mount (2000) listed 13 factors in therapeutic intervention countering spiritual pain experienced by terminally ill patients. These authors emphasize the importance of the therapeutic relationship, establishing contact, respecting the patient’s otherness, effective control of symptoms, obtaining the so-called “clinical biography,” and the subjective significance of the illness in the eyes of the patient and their family. The purpose of these procedures was to properly examine what it is that gives meaning to the patient’s life, to help him or her redefine hope, identify the sources of fears, especially regarding the uncertain future. The authors described spiritual pain of the terminally ill as a suffering connected with being alienated from the deepest levels of the mind. It is tantamount to becoming detached from one’s resources which give meaning to such concepts as hope and goals that are worth pursuing. Understanding spirituality is an important aspect for properly conducted healthcare and clinical practice and clinical practice (de Brito Sena et al., 2021).

## 6. Discussion

Spirituality manifests itself in an individual’s search for meaning and purpose in life, which may, but does not have to, be related to religion. This results in the attachment of a specific system of worldviews, values, and/or religious beliefs that give meaning to life, inspiring and motivating individuals to realize their potential. The spiritual realm brings faith, hope, peace, and strength. Its effects are joy, forgiveness of oneself and others, and awareness and acceptance of hardship and mortality, increased sense of physical and emotional well-being and the ability to transcend the weaknesses of existence by strengthening coping mechanisms (Tanyi, 2002).

There is a real necessity to include spiritual care in the process of patient care, with particular emphasis on geriatric patients. Including the patient in the process of relating to what is outside of him or her, to what he or she feels connected with and belongs to, is an important element in establishing the patient’s individual identity. A careful assessment of spiritual needs is a prelude to a holistic therapeutic intervention. There are two stages of such intervention. The first, important aspect is that the support person becomes familiar with their own spirituality. Knowledge of the foundations of one’s own spirituality and insight into its sources facilitate the adjustment to someone else’s spirituality. Secondly, there is a need to find a way to properly collect data on how the assisted person looked for and found spiritual content important to them. To this end, conversation and dialogue aimed at understanding the other person is of great therapeutic significance. Listening to the narrative of another person, in which he or she presents the story of their life and its meaning, helps at this final stage to see the achievements and values that guided them. The aim is to capture the inner world of the assisted person without any interpretative bias. The life review is never 100-percent positive, hence the attitude of forgiveness is of great importance. The possibility of experiencing forgiveness from others is at the center of the subject’s healing powers, at the same time strengthening their spirituality. The discussed issues show the aspects of the sense of fulfillment that correspond to the definition of mental well-being in old age proposed by Young et al. (2009), which says: *it is a state where there is a psychological and social mechanism of compensating physiological limitations, thanks to which the individual achieves a sense of satisfaction and a high subjective assessment of the quality of life, as well as a sense of fulfillment as a person, present even in disease and disability.*

The study of the spiritual sphere in the context of medicine and social sciences has a long history; many research approaches have been developed to explain this issue. Researchers emphasize the need to unify the concept of spirituality in order to be able to refer to it in the conditions of progressing secularization. In the light of the above remarks, it seems justified to treat the spiritual sphere as a unifying factor underlying the overall philosophy of man. In the context of the interpenetration of cultures and traditions, it is necessary to apply a uniform ethical and spiritual message in clinical practice. What is needed is a universal, practical pattern of perceiving spiritual needs that would be suitable for individual characteristics.

Framework outlined in this study can be used to further investigate spirituality in the context of its beneficial effects on health. It can be assumed that the spiritual sphere provides a mental attitude that promotes health through specific actions (behavior) or lifestyle and approach (emotionally calm/balanced). When things take an unfavorable turn for the individual, it seems that spirituality helps us accept adversity and cope with changes. Research suggests that this type of spirituality promotes a positive, calm, peaceful, harmonious state of mind, self-confidence through connection with the divine that gave life meaning, purpose, and hope. From an intrapersonal perspective may well provide us with coping resources or a reserve that that we can use in times of need. Therefore, to properly understand the benefits that adult patients can derive from established value or belief systems, researchers and practitioners must actively examine the contents of these systems in a respectful way.

## Author contributions

BS, EB, AB, and DK contributed to conception and design of the study. BS and EB wrote the first draft of the manuscript. EB and AB edited the first draft. All authors contributed to the article and approved the submitted version.
